# Supplementary material for: An Investigation of the Nature of Fear within ACL-Injured Subjects When Exposed to Provocative Videos: A Concurrent Qualitative and Quantitative Study
Source: Sports (Basel). 2022 Nov 18;10(11):183. doi: 10.3390/sports10110183 (PMC9692454; doi:10.3390/sports10110183)
Supplement: Supplementary file 1 [file sports-10-00183-s001.zip › sports-1997232-supplementary.pdf]

Supplementary Material

Video S1: Running: (https://youtu.be/zr6NpqrDpQk);
Video S2: Cut and Pivot: (https://youtu.be/3zCg5ifP2dY);
Video S3: Cut and Pivot + Feigned injury: (https://youtu.be/LyTV9UqswMc);
Video S4: Series of Traumatic injury videos: Available on request to main author.

Participant injury and sport: Table S1

Participant	Pre-injury Sport(s)	Level of Sport*	Injury occurrence	Post-injury Sport(s) & level	Year(s) of injury	Return to Sport Rating[#]
P1	Football (Australian rules) and basketball	School	Football competition	Cricket, amateur club level	2016	2
P2	Basketball	National/ International	Basketball competitions	Basketball, amateur club level	2017, 2018	2
P3	Football (soccer)	Amateur/club	Football training	Nil	2016	0
P4	Hockey	Amateur/club	Hockey competition	Hockey at amateur club level	2016	3
P5	Ultimate Frisbee	State	Ultimate Frisbee competition	Ultimate Frisbee at State level	2017	3
P6	Netball, skiing	Amateur/club	Skiing (first), Netball training	Rowing at amateur club level	2011, 2016	2
P7	Cricket, mixed netball	Amateur/club (cricket), social (mixed netball)	Mixed netball game	Cricket at amateur/club level (only)	2017	3
P8	Skiing (moguls)	National/ International	Skiing training	Casual skiing (non-competitive)	2011, 2012	3
P9	Netball and basketball	State	Netball competition	Netball – State level (only)	2015	3
P10	Netball	Amateur/club	Netball competition	Nil sport (runs, gym for fitness)	2015	1
P11	Basketball, skiing	School, casual	Motorcycle accident	Nil sport, still skis	2008	2
P12	Netball	Amateur/club	Netball competition	Nil sport (gym for fitness)	2008	1
P13	Football (soccer)	Amateur/club	Football competitions	Nil sport (gym for fitness)	2006, 2009, 2013	1
P14	Skiing	Casual	Skiing holiday	Nil sport (gym for fitness)	2019	1

P15	Ultimate Frisbee	National/ International	Ultimate Frisbee competition	Ultimate Frisbee at amateur/club level	2019	2
P16	Tennis, netball	School	Accident at school	Nil sport (gym, running for fitness)	2006	1
P17	Lacrosse	Amateur/club	Lacrosse competition	Nil sport (bicycle, gym, hiking for fitness)	2019	1

* Level of sport was identified as either: 'social' or 'casual' (non- competitive), 'school' (playing in inter-school competitions), 'amateur/club' (amateur clubs playing in a local league), 'State' (eligible to represent an Australian State such as Victoria or NSW), or 'National/International' (eligible for inclusion in a national level team with opportunities to play internationally).

# Return to previous level of function scoring: 0-3 where 0= no return to any exercise, 1= Return to basic exercise (gym etc.) but no or minimal sport (i.e. non-competitive), 2= Return to sport at a lower competition level, 3= Return to previous level of sport.

Linear mixed-models analysis: Table S2:

Linear Mixed Models for Fear and Distress for the Four Levels of Threat (CI=confidence interval)

**Fear**	
	Co-efficient (95% CI, p value)
Running	Reference
Cut & Pivot	1.0 (0.4 – 2.0, 0.041)
Feigned Injury	4.1 (3.1 – 5.0, <0.001)
Injury	5.6 (4.7 – 6.6, <0.001)
Random Effects Estimate = 2.1 (95% CI 0.9 – 4.9)	

**Distress**	
	Co-efficient (95% CI, p value)
Running	Reference
Cut & Pivot	0.9 (0.0 – 1.9, 0.053)
Feigned Injury	4.2 (3.2 – 5.1, <0.001)
Injury	5.7 (4.8 – 6.7, <0.001)
Random Effects Estimate = 2.2 (95% CI 1.0 – 5.1)	

Transcripts: Text S1

Participant 1:

Interviewer: Okay. All right. Well, thanks heaps for that. I'm just going to just play a few short videos if that's all right. So I'm just going to show you a series of videos of people performing movements that increasingly load their knee, if you are feeling overly distressed by this, let me know and I'll stop the video immediately. I'm going to ask you how you feel about this video and to rate your levels of fear and distress. Does that sound okay?

P1: okay, all good.

Interviewer: If you can just play the first one, watch it all the way through. How did you feel about video?

P1: Yeah, I didn't feel any like stress or fear at all from that. I really didn’t have too much fear.

So like a zero for fear

Interviewer: how about distress?

P1: Like a zero for distress

Interviewer: Okay how about the next video

You didn't have any feelings about that video?

So how would you rate your fear 0 no fear at all to ten extremely fearful.

P1: Well like a one

Interviewer: What about how distressing zero no distress to 10 extremely distressing.

P1: Still around a 1

Interviewer: Okay next video.

P1: That one was a 7 or an 8.

I think so an eight for distress and a seven for fearful. Yeah. Yeah.

Interviewer: How do you feel about watching that one?

P1: even just seeing someone go down and clutch their knee is the big thing that you see when someone does an injury, like an ACL so yeah the memories come flooding back.

Interviewer: Okay. So just before you watch the last one you can watch just as much or little as you want, okay?

P1: Umm, Its probably the same actually about an 8 for both

Interviewer: 8 for how fearful and distressing out of ten?

P1: yeh

Interviewer: Yeah. Why is that, how do you feel about that one?

P1: um like both of those last two videos. Yes just the action of that knee moving sideways or I feel like seeing someone holding the knee for me is more distressing because I remember that' what I did as soon as you feel it you go to hold it. Yeah.

Interviewer: Thanks. Have you any other thoughts or comments from the interview.

P1: I tried to get it as well as I could. Its hard to talk about it I guess.

Interviewer: are you feeling all right now?

P1: In the moment I would have been able to tell you what I was feeling it's hard to like think back to those problems. Obviously you don't want think back too much to them as well.

Interviewer: Yeah it is a bit hard when you kind of think back?

P1: Yeah, like just the other day because I finished year 12 we were able to open like time capsules. So it was like a letter that I wrote in year 7 to my future self and like literally a whole chunk of it was just

about my ACL and like how I wasn't able to do PE classes and Bill this teacher at the school that had done three ACL’s, he was helping me out but like reading that like made me cry quite a bit. It was just so much detail and just took me back to yeah, how much back then when you're going through it. It's like you're very much consumed in it. Yeah, so that was hard to read.

Interviewer: Do you remember back then were you sleeping and everything?

P1: Yeah, I was sleeping. But I do remember some nights just crying in bed, like not too many… because as I said before playing sport every day doing it because I love it so much and not being able to do it. Yeah, I definitely was crying in my bed some nights.

Interviewer: Sorry. Okay. I'm just going to stop the recording.

Participant 2:

Interviewer: I'm just going to show this four 30-second videos if that's all right. They're just activities increasing the load on your knee. I'll just ask you how you feel about each video and then ask you to rate the level of distress, 0 to 10, and then also the level of fear, 0 to 10, 10 being extreme fear and distress, Just put them in the chat, so you just click on the first video.

P2: Do I have to watch them right now. Is there any chance I can watch them later and send these to you that way? Is that possible?

Interviewer: The fear, and just give me a rating, 0 to 10, of how fearful, and 0 to 10 of how stressing. [crosstalk]

P2: Can I do that?

Interviewer: No problem. That will be fine. Hello?

P2: Sorry. I knew this would happen. This is why I'm stressed, right, the internet's coming in and out. Oh God, can you hear me?

Interviewer: I can hear you.

P2: Is it okay if I watch these later? I don't think my internet is going to hold up right now.

Interviewer: They're all about 30 seconds. Do you want to try or we can just do it later and you can send me them? It's up to you. No, your internet is definitely not going to hold up, is it? [chuckles], lets try it?

P2: Video 1 is a zero fear and distress

P2: Video 2 is a zero for fear and distress

P2: Video 3 Six for fear and six for distress

P2: Video 4 Ten and Ten, Honestly I couldn’t keep watching after the first rebound, it made me feel super sick and distressed

Interviewer: All right.

P2: Thank you.

Interviewer: Thank you, do you need to leave? I will follow up to ensure you are okay tomorrow.

P2: Got to go, thanks Bye.

Interviewer: Bye.

Participant 3:

Interviewer: I'll just show you a series of videos, they're people performing activities and it basically involves their knees. If you're feeling overly distressed by this, let me know and I'll stop the video immediately. After each video, I'll ask how do you feel about the video? and then to rate your level of fear, from 0 being no fear at all, to 10 being extremely fearful. For distress, so 0, no distress at all to 10, extremely. Does that sound all right?

P3: Yes. Sounds good.

Interviewer: I'll just post them in the chat, ask you to watch just the first videos. Number one, if you watch the whole way through and then just tell me how you feel about it. Just how you feel about it

P3: Okay.

P3: How do I get back? Go back now?

Interviewer: How do you feel about that one?

P3: Yes, I'm pretty comfortable with that. The turning, I guess is the thing that would worry me about that a little bit. All right. Yes. I guess that is a little bit in my mind, "Oh yes, I'm about to" because that's obviously part of the rehab when you start to turn and pivot and all that stuff. Yes, that is slightly worrying but that is second nature now, I guess.

Interviewer: If 0 to 10. 10, extremely fearful and 0, no fear at all, where would it lie?

P3: Probably somewhere in the middle, four or five.

Interviewer: Yes. What about distressing, 0 no distress at all to 10 extremely distressing.

P3: Yes, probably four or five again mate, just because that's pretty well how I did it. [laughs]

Interviewer: Yes. Okay. Next video?

P3: Yes, sweet.

P3: Yes, so that's probably a little bit more stressful to me. That movement, that's when I would be more worried about it, going outwards and potentially hurting.

Interviewer: Yes. 0, no fear to 10, extremely fearful?

P3: Like seven-ish.

Interviewer: Seven-ish, yes. What about distress? 0, no distress to 10, extremely distressing.

P3: Yes, probably around seven, mate.

Interviewer: All right, video number three.

P3: Cool.

P3: Yes, that'd be pretty bad, [laughs] high, like eight or nine, I guess.

Interviewer: How fearful.

P3: How fearful of that happening, that would be pretty bad. I would change it [laughs]

Interviewer: Yes. What about how distressing?

P3: Yes. About the same mate, 8 or 9

Interviewer: Yes, but how did you feel about watching that one?

P3: That's pretty well what I was doing at the time, just nothing bad at all, and then went to turn and my knee went one way and the rest of me went the other way. That's pretty bad. Is this the last one?

Interviewer: It's the last one, yes.

Interviewer: Oh

P3: Oh, Fuck me. [laughs]Oh man, the basketball one is fucking atrocious. The one of the soccer player, is that Joe Cole? I think that's fairly similar to what I did. That would be pretty bad. I'd say, 9 or 10 there and 9 or 10 distress as well. Yes.

Interviewer: Yes. How do you feel when you watch that kind of stuff?

P3: It makes my knee hurt a little bit.

Interviewer: Does watching that bring about similar feelings when you hurt yourself?

P3: Yes, definitely. Especially the soccer one because that is almost identical to what I was doing.

Interviewer: Yes. Okay.

P3: It's pretty well out of nothing just [laughs].

Interviewer: Just went down. Yes.

P3: Yes.

Interviewer: All right. Well, thanks a lot for participating today, that's really interesting stuff. If you have any other thoughts or any other comments or anything about this study?

P3: I think with the distress or how worried I am or was when I did it, a lot of that was already built up because I'd seen it happen a lot. If that makes sense. If I didn't know nothing about it, or hadn't seen it happen, or had people that I knew do it, it probably wouldn't have been as bad for me, I think. I think about that a fair bit then I just worry about it more than I should. Does that make sense?

Interviewer: Yes, it does.

P3: Definitely, working at the snow didn't help in that regard because they're constantly going on about these positions not to get in and they always give us knee checks and all that stuff. It happens to 5 to 10 people a season, like ski instructors, snowboard instructors so it's super common so you know. I knew how bad it was so I was like," Oh, fuck, now I'm one of those people, that [laughs] that it's happened to." I was like, "How have I worked at the snow and gone into the snow my whole life not done anything, and now I've gone back and played soccer and fucking fallen over and done it."

Interviewer: Yes. There are interesting aspects there it will be interesting to see how you go, on going and see if you've gained some of that confidence because you've said it. I will just put in my two cents. You said it's something you really enjoy something you're really passionate about. It's that way. If you've never actually hurt it at the snow. It's just a really interesting.

P3: Yes. I think I'd be okay, to be honest. I think if I just went down for a couple of days I think I'd be all right.

Interviewer: Do you think if you expose yourself to it, it would help you get over the fear?

P3: I think so. Yes, definitely, you're right, yes.

Interviewer: Do you think the reason why you're not going is you are? Now you're reflecting on a little bit?

P3: Yes. Probably a little bit. It's just a few other factors like I haven't been down there and stuff like that as well. That's not the defining thing that I haven't been back for a while. Yes, it's definitely one of the reasons but I'm pretty sure I'd be fine. I'm pretty confident in my ability for that. I know the movements and I know how my body works on a snowboard, so I think I'd be okay with that. I feel I can control my movements pretty well on a snowboard. I feel a lot more confident and now I think the soccer thing is that it can just happen so easily just your foot goes the wrong way and then all of a sudden there, it's done.

Interviewer: Just last, little bit, two cents from me. I worked in Japan for six months or five months, and there some serious knee injuries on snowboards were about 2% versus skiers were about 30%, so a lot. Just because you have that third plane of movement you have on the skis. I think day one, getting apart, sitting on the snowboard hitting a few big lines big rows.

P3: Yes well, I was a snowboard instructor, not a skier, so I won't to be down on skis. [laughs]

Interviewer: Thanks for doing the interview. I really [crosstalk].

P3: No worries, mate. [crosstalk] if you need anything else.

Interviewer: Cheers. That'd be amazing. All right.

P3: Thanks mate. Cheers.

Interviewer: Have a nice night. God bless.

P3: You too mate. See you.

[END OF AUDIO]

Participant 4:

Interviewer: Thanks for this. Great. There are some really interesting insights there. Do you mind if I show you a few videos? They're all about 30 seconds.

P4: Yes.

Interviewer: They're just a series of videos of people performing activities that increasingly load the knee. If you're feeling overly stressed by this just let me know and we'll stop the video immediately. After each video I'll ask you how you feel about it and then also to write your levels of fear 0 to 10; 0 no fear at all, to 10 extremely fearful. Then distress; 0 no distress, to 10 extremely distressing. Does that sound okay?

P4: Okay. Yes.

Interviewer: All right. I'm just going to place it into the chat on the side. I'll get you to just watch the first video, and just watch it the whole way through, and then let me know how you feel about it.

Interviewer: Did you press the first one?

P4: It's funny with one-- What was the first one? I'll get back to that,

Interviewer: Just watch the first one. Let me know how you feel about that first.

P4: The guy, the basketball one guy?

Interviewer: Just the straight running one. The number one on the chat menu.

P4: I've got a different-- That maybe it. I'm pressing the wrong thing last time.

Interviewer: Just have a look at the first.

P4: Sorry, Ill watch the first one:

P4: That really doesn't bring up any issues at all.

Interviewer: How would you rate the fear 0 to 10?

P4: Zero probably, I think.

Interviewer: The distress?

P4: Definitely a zero.

Interviewer: All right, cool. Go to the second video.

[pause 00:46:39]

Interviewer: How do you feel about that one?

P4: It was all fine until those little lunging forward before they turn**.** That movement was pretty much exactly how I did mine, so the fears not huge but something like a two. It's still relatively controlled and distress a zero still. [crosstalk]

Interviewer: All right. Next video?

[pause 00:47:47]

[background noise]

P4: Other than it being funny. [laugh]

Interviewer: What do you feel about that one?

P4: I don't really know how I feel about that one. I feel okay about it. It doesn't grab me like the first one, but doesn't really-- Yes, I feel okay.

Interviewer: How do you rate the numbers up for you?

P4: Fear would be Maybe a one.

Interviewer: The distress?

P4: The distress, yes, a one. Yes.

Interviewer: The last video?

P4: I already watched it.

Interviewer: How do you feel about that one?

P4: In the middle of it, on the basketball court one or the netball court at the school, that one I found the most distressing. Otherwise, I'd say I found it amusing in a weird way. [laugh] Kind of gross, but I just separate in terms of elite sport and maybe why I found the basketball one maybe a bit more. Fear-wise a two and the distress probably a one or a two. It felt pretty gross, some of it.

Interviewer: In regards to this, what does distress mean to you as we're defining it right now?

P4: Like upset. Fear would be you're scared and the distress would be- it means I'm really upset and heightened emotion around that, yes.

Interviewer: You think you mentioned that one of them reproduced some of the feelings that you got. Watching the video, you felt some of the things that you experienced when you hurt your own knee?

P4: I just think it's more relatable in that, I guess, elite athletes when they do an injury,-- I know it's their entire life, but that they do have an entire team of people straight away onto it doing everything for them to help them out. When its someone a poor old pleb that Doesn't realize how much of an impact it might have currently, so, yes.

Interviewer: Thank you so much for today. Do you have any comments or thoughts around anything in the interview, with the chat? Sorry, we ran quite long.

P4: It's okay I'm a waffler. I don't really know how to answer some of them. Some of them, I've never really consciously thought about these things. It was interesting to think about it in a different way.

Interviewer: It should be interesting to have a little bit of reflect-- [crosstalk]

P4: Is my friend--

Interviewer: Yes?

Interviewer: Have a great night. Cheers.

P4: See you.

[END OF AUDIO]

Participant 5:

Interviewer: Yes. All right thanks for that. That was good. I'm just going to show you a series of videos of people performing activities that increasingly load the knee. If you're feeling overly distressed by this let me know and I'll stop the video.

After each video I'm going to ask you how you feel about this video and to rate your levels of fears as zero no fear to 10, extreme fear and then also distress so zero no distress to 10, extreme distress. Does that sound okay?

All right let me grab these four. I'm just going to pop them up in your chat window, they're about 30 seconds each. I'll get you to watch the first one all the way through and then let me know how you feel about that.

P5: The fourth one is the injury one, right?

Interviewer: Yes, so just the first one first. Are you happy watching these?

P5: Yes, I'm happy watching the ones that aren't the injury ones.

Interviewer: All right. We'll watch the first two then.

P5: Okay.

Interviewer: Just watch the first one and the second one.

P5: First one, zero. [laughs]

Interviewer: Zero. how fearful? Zero to 10.

P5: Yes, zero. [laughs]

Interviewer: What about distressing? Zero to 10.

P5: Zero.

Interviewer: Any other thoughts or feelings on that video?

P5: It looks very easy.

Interviewer: Next video, number two.

P5: Probably also a zero on both.

Interviewer: No problem. Zero for fear and zero for distress.

P5: Third video, Five for fear and Four for Distress

Interviewer: Any other thoughts or feelings on that video?

P5: No

P5: Fourth video, Five for fear and Five for Distress

Interviewer: What kind of feelings and emotions do you have when you are confronted with a video that depicts an injury or ACL injury?

P5: I find that they just pop into my brain randomly and it makes me really uncomfortable because you've got to try and get it out and think about something else which is why I really hate watching them because I don't need-- I've seen enough in general of all sorts of random things not even just

ACL injuries, just random injuries. I just don't want new fresh ones that decide they want to come in every now and again.

Interviewer: Yes. When they pop into your head, is this something that's been-- Is this something that's been happening throughout the experience, the injury experience? Have you had recurring issues of--

P5: It could be anything like stepping on broken glass or something like those sorts of things will sometimes pop in and I'm like, "That's got nothing to do with my injury." It's just something bad that could happen which I guess actually--

[silence]

P5: I think since the injury I've been more aware of things going wrong in day to day like, falling down the stairs or something. I was like "You could really hurt yourself if you fell down the stairs."

I think that has been a bit more present since the injury. It will pop up into my brain more often, the potential for injury in everyday life. What was the question again? I'm sorry. [chuckles]

Interviewer: That was the question. Have you ever had any issues with sleeping or any unwanted thoughts or anything in the mellow times?

(P5 headshake)

Interviewer: No, Overall the ACL injury experienced, what do you think it's done in terms of changing your life or sensitizing you to certain things?

P5: I think it's made me more sensitive to the potential of injury.

Interviewer: How do you feel about that? What would be the net effect of a future injury? Why is that so important to you?

P5: I think it's just so frustrating not being able to do things that you want to do like everything. [chuckles] Future injuries could potentially see you bed-bound for a month or it could just mean that you just don't get to play sport for the next two weeks.

Either one is frustrating because it's stopping you from doing something that you want to be doing and so I guess, you just try to avoid it. In terms of the avoidance, you are going to keep playing some sport though. Where is this line of avoidance versus participation in terms of your thoughts and feelings about your injury?

P5: That's where I'm confused as a person because I think about this stuff a little bit, enough that it’s different from what it was before but I refuse to-- I'm not going to stop playing sport. I have way too much fun and you can prepare yourself. It’s just there like sitting in the back of your brain like, "I'm not going to stop doing fun things because I'm worried about it." But you are worried. [chuckles]

Interviewer: Last question. You mentioned self-talk but is there any other things we touched on, was there any other things that have helped you think in dealing with psychological issues except at the gym obviously getting strong?

P5: I think I just had a very supportive community coming back as well. There was never any pressure to do things at 100% from the get-go. People knew that you're always coming back and so it was, "Just do as much as you can no one's going to judge you for it. We know that you are worthy of being on this team no matter what level you're playing at."

It was very easy to take it at my own pace and just come back and get comfortable and then keep stepping up the intensity. It wasn't stressful like, "Oh, I’m back so I have to be at 100%.

Interviewer: That's some great messages from people around you. All right. Nice. Thanks so much for that. I appreciate the time. You have a very interesting experience and interesting story. I love the fact that you understand it.

You understand that there's things involved and you're obviously still a bit worried about it, but you're still going to give it a bit of a crack. Any thoughts or feelings or anything after having a chat about it?

P5: I feel like yes, it makes me think of more about why I do things a certain way oh you see why I think about same things but not really.

Interviewer: Do you think with the physical stuff you've mentioned that you think are pretty good physically, but you need to maintain it in so much. Do you think that that will at one point go away and you'll be at a physical level where you don't worry about it anymore or do you think the worry-- Because you mentioned you think you're pretty physically good, but then it's not quite as good but you still going to be ongoing worried about it. Is there a point where that worry will stop?

P5: I think once I've settled back into my gym program and working everything out, I think that's when worry goes away. When I'm in the peak of my season, there's little to no worry. It would just be the muscle wastes away so quickly now just because of the injury it just happens. Compared to the other leg, it just gets smaller so much quicker.

It's just when that happens I think the worry really comes in whereas once I've got everything back up and I'm going to the gym and keeping it maintained at the level that I need it to be, I think the worry goes away.

Interviewer: That's the biggest thing, isn't it? All right. Thanks, heaps. Yes, very interesting. I hope you get back to everything. You're already obviously doing so well, so maybe great athlete going.

P5: It's been three years. I hope I was doing okay. [chuckles]

Interviewer: Good and just when you go to the physio also have a chat through any uncertainties or anything else that you've got. Yes, that's good. All right. Thanks so much for that. We've got another part of this same project next year sometime. Would you mind if I keep you on the email list for anything possibly get in touch.

P5: Yes, sounds good.

Interviewer: Amazing. Have a great day at work. Thanks for taking the time, sorry we ran a bit long as well. Apologies.

P5: It's all good. Have a good one.

[END OF AUDIO]

Participant 6:

Interviewer: I'll show you a series of videos of people performing activities that just increasingly load the knee. I'll ask you at the time how fearful the videos are, zero being no fear at all to 10 extremely fearful, and then I'll ask you how distressing they are. Zero, no distress at all to 10 extremely distressing. I'll just ask how you feel about it. If you feel uncomfortable at any time, just let me and we can stop the video. You don't have to watch through any of them. Okay?

P6: Sure.

Interviewer: Sound good. All right. I'm just going to attach the videos into each app menu. Has that come up, yes.

P6: All right. The first one?

Interviewer: Just get you to watch the first one all the way through and then tell me how you feel about it.

P6: Yes. I feel fine about that one.

Interviewer: Yes, so how fearful are you zero to 10? Zero no fear, 10 extremely fearful.

P6: Zero.

Interviewer: How distressed are you? Zero, no distress, and 10 extremely distressed.

P6: Zero.

Interviewer: Okay. Next video.

P6:

Zero again in terms of how fearful I was, and zero for distress as well.

Interviewer: How do you feel about that video?

P6: I think I was fine.

Interviewer: Okay. Video number three.

[laughter]

How do you feel about that one?

P6: What were the two things again?

Interviewer: The first is how do you feel about watching that video?

P6: Not great.

Interviewer: Okay, in what way?

P6: You seem to be like, "Oh, that hurts." It's like you remember that pain watching it.

Interviewer: So how fearful was it 0 to 10?

P6: Six.

Interviewer: Six, and how distressing was that, watching that, 0 to 10?

P6: Four.

Interviewer: Four, okay. The last video, video four

Interviewer: How do you feel about that video?

P6: It's a little bit uncomfortable watching it.

Interviewer: In what way do you feel uncomfortable?

P6: I don't know. You feel like all your muscles seize up and go like, "Oh no don't do that". I didn't love that one. When I start watching the whole succession it kind of does end up a little bit comical. It does make you feel uncomfortable.

Interviewer: How would you describe that uncomfortableness that you're feeling?

P6: Probably like my body tenses and I sort of imagine that that's your knee and then your muscles will kind of tense up unconsciously to protect it, which is good, good little body. That's how it feels.

Interviewer: What would you rate your fear on that video 0 to 10, 10 being extremely fearful, 0, no fear at all?

P6: Maybe like a six.

Interviewer: What about how distressing?

P6: In terms of between that one and the last video, the last video I probably thought of it being myself a bit more, whereas that video wasn't, I sort of fear for them but not as much for yourself.

Interviewer: How distressing was that last one 0 to 10, 10 extremely distressing?

P6: Maybe that one was like a 5 or 6.

Interviewer: Did any of these videos, you just mentioned it, but did any of these videos bring about similar feelings or emotions than what you've experienced during your ACL injuries? Watching them that is.

P6: Yes, that like, "Oh, that's going to hurt and be annoying for you for ages". That sort of feeling comes back. Not sure anything else. All the disappointment of like, "Oh".

Interviewer: All right, thank you. Do you have any other thoughts about the chat today?

P6: No, not really. It was interesting.

Interviewer: All right, one sec.

[END OF AUDIO]

Participant 7:

Interviewer: These will just be of people performing activities that increasingly load the knee. If you feel a bit distressed by this just let me know and I'll stop the video immediately. For each video, I'm going to ask how you feel about the video, to rate your levels of fear and distress. Are you ready to proceed?

P7: Yes.

Interviewer: In the chatbox, I've just posted them through. You've just got to click the top one. If you see it in the chat and then just play it. Play it through. It's about 30 seconds and then I'll just ask you how you feel, your fear, or how your levels of distress and levels of fear are 0 to 10.

Interviewer: How do you feel about that one?

P7: No fear.

Interviewer: Okay. Any distress?

P7: No fear at all. Absolutely not.

Interviewer: Next video. I haven't divided them very well.

P7: That's okay. Plant and cut?

Interviewer: Yes.

P7: For this one now, absolutely no fear or no distress. Before I started the activity again, that was one thing that I was extremely wary of. Now having done it, I have full confidence in my body to be able to constantly do that again.

Interviewer: So before you start activities, if 0 was no fear at all to 10 extremely fearful, where do you think you would have been on the scale?

P7: Before I started activity? It would have been probably a seven post having done that in a non-modified environment like in a game or something, zero.

Interviewer: What about how distressing 0 no stress to 10 extremely distressing before the game [crosstalk]?

P7: To watching the video, or-?

Interviewer: Yes.

P7: -before doing it. Watching the video now, I don't have any real distress, no distress watching it, no.

Interviewer: Okay. Next video.

P7: This is good. I like this.

Interviewer: How do you feel about that video?

P7: Yes. To be honest, it just makes my knee hurt. [laughs]

Interviewer: How fearful--?

P7: I do have some fear of doing an activity like that and that happening again. I would say some fear. It would probably be a 5 out of 10.

Interviewer: How distressing?

P7: If I was to watch that before having played sport again, it would be a 10 out of 10. If I watched that, and I think that's probably just like a contextual kind of thing, if I had watched that before I had done anything like that before, I probably wouldn't want to play any of the sports. Now that I've actually done a lot of that already since having my surgery, I know I've got confidence in my body because I've done those things, but I still have some fear of being re-injured. That's a good video.

Interviewer: 5 out of 10, fearful. What about how distressing, 0 no distress to 10 extremely distressing?

P7: Yes, probably about five in terms of distressing, I just feel bad for them knowing what they now have to go through.

Interviewer: I think you may have just watched the fourth video. I think maybe there might be another link just one up. What video was that one? Was that the plan and cut or was it was plus injury, or was that the--

P7: No, that was-- Oh, no, now I've got the Alex Rance type of cut. I was watching this one at the time, I felt really sorry for him at the time because I knew what he had done.

Interviewer: How fearful would you say that is 0 to 10?

P7: Fearful, I'm probably less fearful watching this one but I just have so much like more of the level of empathy. It's more distressing watching this one for a weird variety of reasons. For him he was in real agony. He's really really good, and yes, it's just a real shame. That's probably a seven in terms of distress and a five in terms of fear.

Interviewer: Have you had any other thoughts around the questions today or around your thoughts or feelings around your ACL experience?

P7: No. I thought I was really lucky in terms of the services that were available to me. I didn't have to wait for surgery, got it in a private hospital. I worked at the hospital that I had my surgery at so I was treated really well. I had a great relationship with my physios and I still have a friendship with them to this day. I had a great support network. Family were great, friends were great. I know not everyone has the same experience with knees or with any injury or any trauma. I was pretty lucky, or I think I was anyway.

Interviewer: Okay. Good. Thanks so much for the time today. That's helped a lot.

P7: No worries, mate. No worries.

Interviewer: I appreciate it. I'm just going to stop the recording on this one.

[END OF AUDIO]

Participant 8

Interviewer: All right. If it's all right with you, I will show you a series of videos of people performing actions. Just going to increasingly lower the knees. If you're feeling overly distressed by this, let me know, I'll stop the video immediately. After each video I'm going to ask how you feel about this video, to rate your levels of fear and stress. Zero being no fear to 10, extremely fearful. Then also fear and then distress zero, no distress to 10 extremely distressing. Does that sound good?

P8: Yes.

Interviewer: I'm just going to post them into our little chat box. What you do is watch the video the whole way through the first one and that's it. Then just let me know how you feel about it [crosstalk]. That first YouTube link, yes.

P8: Can you hear me?

Interviewer: Yes. How you feel about that one?

P8: 10 is the highest for discomfort, right? It's probably a four. As soon as they cut back, if I knew I was going to cut back I'd be thinking about it but would I be afraid to do it? No.

Interviewer: How fearful from zero to 10, sorry?

P8: Four.

Interviewer: Four. How distressing, zero to 10?

P8: Probably two.

Interviewer: Two. Awesome. Next video.

Number two.

P8: On the phone, it's going to bring back the bloody-

Interviewer: Do you want me just to send it again, you got it?

P8: Now I got it. It's a separate section. I got it.

P8: That's probably just a little bit more than the last. As far as the context, I have to put in it, as if I knew I was going to have to do that. I suppose discomfort had to go up to a five because I'd have to really think about it a little bit more and leading up to it. Is this going to hurt, is my knee up to it and I suppose level of distress is two or three.

Interviewer: Fear was a five and then distress was a two or three?

P8: Yes.

Interviewer: Great. Last two [crosstalk].

P8: I'll do the last-- does it match up to the last video to go?

Interviewer: There's only two 30 second clips left.

P8: Okay, since she's coming back, I'll watch this next one.

Interviewer: Yes.

P8: Oh yes you got four clips in total.

Interviewer 2: Hi.

P8: Yes. How you doing? Sorry, sorry just hold it there

P8: I am just watching that 3rd video, I better be quick because I think they are about to wheel me through I think they are running early.

So I think for the 3rd one not to dissimilar to the 2nd I don't think it causes me much more distress or fear watching someone have a knee injury performing that kind of movement.

Interviewer: how would you rate that fear

P8: This new video I think about a 2-3 for fear and 5 for discomfort.

Interviewer: how about the last video.

P8: there's some pretty good injuries there. The last one is pretty epic, I suppose the distress is probably up to a 6, but the fear is still probably a 2 or a 3.

My view on sports injuries that they are really part and parcel. If you want to play sports and push it hard you have got to expect that at some point something is going to go wrong. I don't have a fear of having a specific injury. I will rephrase that, there is a fear that you are going to hurt yourself I think everyone has that, if you don't, some people might not but im sure most people do. Even if you are a big wave surfer you are going to have some fear. The fear might not be related to an injury but it might be more that you might drown or seriously hurt yourself. Like, smash your head on the reef and knock yourself out. This isn't a fear on the high scale which would make you want to stop doing this stuff.

Interviewer: Does watching any of these videos bring about similar thoughts and feelings or emotions to what you experienced during the injury.

P8: no I cant say it has, no

Interviewer: any other comments on the interview today

I massively appreciate the time today.

P8: I have a goal for this today, my goal is to go surfing for the last 10 days in January if I do the rehab right and play my cards right it should work out. I have done the rehab so many times that I know if I follow the right steps it will work out. If I skip steps then it won't turn out as well.

Interviewer: do you think you will go back and have a ski at any time

P8: I reckon I might get some flights to NZ in September before it goes through the roof and gets booked out.

Interviewer: Good luck for today and thanks again for all the time. Cheers Bye

END

Participant 9

Interviewer: Thank you so much for that. So it was all right with you. Can I show you a few videos? These are of people performing activities that are going to increasingly load their knees. If you feel distressed by this just let me know and I'll stop the video. After each video I'm going to ask you to rate your level of fear and distress.

P9: Okay,

Interviewer: Okay

Interviewer: What is your level of fear, zero with no fear or 10 being extremely fearful.

P9: Probably zero. Not too much for you with that one.

Interviewer: What about stress as you watch that video?

Zero no stress at all to 10 extreme distress

P9: 0 again with that one.

Interviewer: You got any other thoughts or additional thoughts or comments on that video?

P9: No, I've remembered running in a straight line. So brings back memories, I think running straight takes away a lot of that fear. I think it's that change of direction for ACL recovery that causes a bit more stress.

Interviewer: So let's proceed to next video this one.

Interviewer: So as you watch this video, what is your level of fear 0 no fear to 10 extremely fearful.

P9: Probably a bit higher so maybe like a 2 just because it is a change of Direction. But I think if you had asked me this during that process it would have been a lot higher it probably would have been like a 8 or something like that. But at the moment because I do change direction and I do a lot of drills like this. I don't know. I feel a lot less fearful.

Interviewer: So what about what's your level of distress as you watch this?

P9: Yeah, probably a one one or two If I had of watched this and done this whole interview during the injury or just after the injury my responses would have been a lot different. It would have been a lot higher. Like even when I used to watch people change direction, it would give me that like, 'uh, can't imagine doing that'. So yeah, it would have been a lot different

Interviewer: Any other comments or thoughts on this one? Alright, let's move on to number three.

Interviewer: Okay level of distress as you watch this video Zero no stress at all to ten extreme distress.

P9: Oh, yeah, six or seven. It's watching injuries still it's just yeah, not ideal like it's not yeah.

Interviewer: Last one

VIDEO 4

Interviewer: So level of fear with watching this video. 0 no fear at all to ten extremely fearful

P9: Probably like seven. Those are some pretty intense injuries.

Interviewer: Yeah, and any distress as you watch those videos.

P9: Yeah, probably seven as well for that one.

Interviewer: not too dissimilar to the video before. Yeah.

P9: No not I think yeah

Interviewer: All right. Thanks so much I am very appreciative of your time. Thank you a lot. Any comments or thoughts around the questions around anything we kind of went through today.

P9: No, it was actually really good to do kind of unpack everything that I do because I have’nt really reflected on it in a long while, it's quite interesting actually, it will be really interested to see how it all unfolds after you finished it. So yeah, thank you for having me. And yeah, I look forward to finding out how it all goes with your research and other people's responses and things like that. So, yeah. Thank you.

Interviewer: You thanks so much.

Participant 10

Interviewer: Thanks so much for that. Now I'm just going to show you a series of videos, just of short videos of people performing activities, just that are increasingly loading the knees. If you're feeling overly distressed by this, just let me know. I'll stop the video immediately. After each video, I'm going to ask you how you feel about this video and to rate your level of fear, 0 to 10, and then distress, 0 to 10. 0 being no fear or no distress, and 10 being extreme fear or extreme distress. That sound okay?

P10: Yes.

Interviewer: I'm just going to send them on the message bar, which you should be able to get out, just down on the bottom of the screen in chat. You should just see a chat button.

P10: Yes.

Interviewer: I'll just resent them, so there should be four. If you click the first one, watch it all the way through whenever you're ready.

P10: I've watched them. Did you want me to watch all of them?

Interviewer: No, just the first one is fine. How did you feel about that video?

P10: That was fine. If I wanted, I could do that. It's not very distressing, or it doesn't invoke any fear.

Interviewer: What would be your level, 0 to 10 of fear?

P10: Probably, about one or two.

Interviewer: Then what about distress, 0 to 10 distress?

P10: Something similar.

Interviewer: One or two, okay. Next video.

P10: Probably, about three or four for fear and probably about two, three for distress.

Interviewer: Any other feelings about that video?

P10: Not really. I probably won't try and do that. That's all.

Interviewer: You wouldn't try?

P10: No.

Interviewer: Video three.

P10: That's exactly what happens when I try to do things like that.

Interviewer: How do you feel about that video?

P10: Probably, fear is about eight, and distress is eight as well. My fear and distress go hand in hand, or maybe I can't tell them apart.

Interviewer: There's one more video. I'd get you to watch that; you can just stop it whenever.

P10: Oh, that looks painful.

Probably about eight or nine again.

Interviewer: Eight or nine for fear, and then eight or nine for distress as well?

P10: Probably, a bit high for distress.

Interviewer: 9 or a 10 for distress?

P10: Yes, probably not as much, just because I now wouldn't try and do those things; that's why it's probably not invoking that fear.

Interviewer: What's the difference in regards to fear and distress then for that last one?

P10: If I look at it, it's distressing versus fear is more like, "Would that happened to me as well?" Like the fear of that happening to me.

Interviewer: That's being the difference between fear and distress throughout or what we chatted about?

P10: I think so, yes.

Interviewer: Any other feelings about that last video?

P10: I don't know how to express it, but it's like, "Ooh." It was distressing to watch them because it looks so painful. A couple of times, while playing, I've ended up in something similar, so it's more like a little bit anxiety as well, just feeling anxious about it because it brings back-- I don't know, bad memories. I distinctively remember falling on the ground and just touching my knee in pain in a similar situation. That's why the inside is tenser.

Interviewer: Thanks. During any of those videos; obviously, that last one, it brings on similar emotions and feelings to what you experience during your own ACL experience?

P10: Yes. Not all of them but a couple of them I could actually very much identify with.

Interviewer: What does that identification mean to you?

P10: It was more like I was exactly in that same situation, and that's probably what I looked like as well to my friends who are watching.

Interviewer: How does that make you feel?

P10: I should have probably gotten this looked at sooner.

Interviewer: Thanks so much.

P10: I think more regret more than anything else.

Interviewer: Thanks for today. That's how it takes. Have you got any other comments or anything else in regards for what we talked about today?

P10: No, not really. You're doing an interesting piece of research because I'd actually-- Even after knowing, you had never actually thought how it made me feel. I was thinking about feeling in the physical sense and not emotional sense. It's interesting what you're doing.

Interviewer: Thank you so much. If it's all right with you, I'll transcribe what we talked about today. Then I'll send it through to you at a later date. You can have a bit of a read of it.

P10: Sure. Anything I can do to help, I am more than happy to.

Interviewer: Just for your own information as well. If you got any other comments or any other thoughts, please, feel free to let me know.

P10: Sure, no worries. When do you finish your thesis?

Interviewer: A long time. [chuckles]

[END OF AUDIO]

Participant 11

Interviewer: All right. Thanks so much for this. Awesome. I just wonder if it's all right with you, to show you a series of videos of people performing some activities that are increasingly loading the knees. If you're feeling overly distressed, let me know, and I'll stop the video immediately. After each video, I'll just ask you how you feel about the video. They're about 30 seconds each. We'll rate your levels of fear, 0 to 10, 0 being no fear and 10 extremely fearful. Then, also 0 to 10 on distressing. Does that sound okay?

P11: Yes. Are these people going to injure themselves or are they just doing the exercise?

Interviewer: The last one will be a bit-- Is that okay?

P11: Yes, that's all right. I'll watch it.

Interviewer: In the chat menu, which will be on the side. Just click the first one, play it through.

P11: Yes, that's all right.

Interviewer: How do you feel about that video?

P11: That's a one. That's okay. That's not--

Interviewer: 0 to 10, now--

P11: Just go a one with that, that was all right.

Interviewer: 0 to 10, how distressing?

P11: I had a feeling there was going to be some sort of turn in there when I saw them running straight. Fearful: maybe one, distressing: maybe two. Just put it two because I knew that something was going to happen. I'll watch number two.

Interviewer: Number two.

P11: I might have watched the same video. Hang on. I keep going to the same. [silence] I think I forgot. Was the first video just a straight run?

Interviewer: Yes.

P11: I think I might have watched video two first. Sorry.

Interviewer: What do you feel about the first video?

P11: Yes, the first video was fine as well. That's just one.

Interviewer: One for fear?

P11: Yes.

Interviewer: How about distress?

P11: Yes. Just one for distress. That's okay. Right, I'll try number three.

That one was all right.

Interviewer: How do you feel about that one?

P11: Still, just put twos. I think watching someone else is a bit different.

Child: [inaudible].

P11: What's up, mate?

Child: [inaudible].

Yes. That was okay.

Interviewer: Two for how fearful and two for how distressing?

P11: Yes.

Interviewer: Then the last video.

P11: Oh, I can't watch these videos.

Child: [inaudible]

Come here then.

Child: Mummy.

[silence]

This one's a good. I'll just go for sixes.

Interviewer: How do you feel about that last video?

P11: I don't like it.

Child: [inaudible]

P11: Down there's fine.

Interviewer: You said it was six. How fearful, 0 to 10?

P11: Yes, fearful is probably six because I knew from the ACL injury hitting that it was going to be yuck.

Interviewer: What about how distressing?

P11: Maybe a six as well.

Interviewer: Do any of these videos bring about similar feelings or emotions to what you experienced during your ACL injury?

P11: Yes. The last one, definitely because I could see it mid sport, whereas the first few were just exercise videos. I don't think I've ever really been scared of playing or just doing normal exercise as much as something in action in some sort of game or actually chasing someone. The sports one's definitely a lot worse.

Interviewer: Okay, thanks for that. Any other thoughts or comments about the chat today?

P11: No, no, it's good. I'd like to see what your results are and see what other people say. You're trying to do some that don't have fear and some that do, or--?

Interviewer: One minute. Sorry. We've got people doing-- this stage is only people with fear, just having a chat. This will be the first study. Then the next studies will actually be scanning people's brain to see if there's a change post-injuries, to see if the way your brain's actually functioning is

different, and that'll be versus normal vertical control, ACL versus ACL. That'll be very interesting as well.

P11: Interesting. I'll have to tell my physio when I catch up with her next time. I haven't even asked what they do at uni and if there's anything to do with mental recovery. I'll have to ask her because I'm interested.

Interviewer: This is a proof of concept study because there is really not much there. This is an early into it, as you'd say. You've got some really interesting stuff going on throughout everything, which is really interesting. I think your guidelines of people that are telling you that they're getting back in six weeks and saying that people are walking in four and stuff like that. I think that's, as you said, before people not taking the piss, or not being rude, but they're just not understanding that that's not actually what happens. When you repair an ACL, they'll get to another injury and be always like, "I was back in two weeks from my shattered thumb." You're just like, "Yes, but that's just something that happens post-surgery."

P11: Yes. It is people that haven't done it themselves and haven't-- It's not the people that have had an actual proper injury, but it doesn't help, people shouldn't do it. It's genuinely people trying to be encouraging, but I think it does more damage than good. Especially now that I've been back on it.

Interviewer: Everyone's got a friend of a friend story that was back at two and a half months and did a backflip.

P11: Yes, pretty much. Well, thank you.

Interviewer: No probs. That was super helpful. Does that chatting about it, help in any way? How you are feeling about it?

P11: No, I want to play again. I want to play again. I'm supposed to be catching up with my physio in a few weeks so I'll tell her and she'll laugh at me for not playing again and then I'll say, "No, I've got mental issues. I've got a mental block and that's why I can't do it." See what she says.

Interviewer: My only advice maybe, just, with her, if you've got a bit of time before the season starts, do some high-level stuff. For you, it's obviously game-related. There's no point in you doing single leg jumps, single-leg landing, bounding because that's not relevant for what you want to do. You need to be like doing game related things.

P11: I need to actually go out and do some coaching. She did make me do that. I was trying to do some with my husband on weekends and stuff. He doesn't take it as seriously either. He's the person that would just go back to sport. I think he's doesn't understand it either but he did tag me in this to tell me to do it. He obviously knows I've got a block but yes, and I do need to, I need to go down and actually do some playing before I actually play.

Interviewer: Just so you can also spend some time just thinking through some of the movements, planning, thinking how it feels when you catch. Just trying to normalize it. Think of yourself in the first person playing the game that actually activates the same areas in the brain as actually going and doing it. It's like a low-level exercise thats completely safe, so you can imagine running, passing, grabbing, and it's just going to be an in.

P11: Yes. I will. It's been long enough. I'm going to play again next year. [chuckles] I will.

Interviewer: Thanks. Nevermind, I might just keep you on the books as well if [crosstalk] next year as well.

P11: Yes, definitely.

Interviewer: Also, sorry it took so long. We blew out of time.

P11: That's all right. I'll go and apologize to my friend chasing the kids. That's all good.

Interviewer: Thanks again. Have a great night.

P11: Thank you. You too. Thanks.

[END OF AUDIO]

Participant 12

Interviewer: People performing activities increase the load in the knees. If you feel uncomfortable or distressed let me know and I'll stop the video. After each video, I'm just going to ask you how you feel and then also to rate your fear 0 to 10. 0 being no fear to 10 being extremely fearful. Then also your distress, 0 being no distress to 10 being extremely distressed, does that sound okay?

P12: Yes.

Interviewer: What I'm going to do is I'm just going to copy into the chatbox. You just play maybe video one first and just watch it through, and then go from there.

P12: Sorry, my internet's very slow, it's just loading now.

Interviewer: That's good.

P12: There's no sound in the videos, is there?

Interviewer: No.

P12: Okay, there's no-- Hang on, what are the questions? The fear is very low, probably like one to two.

Interviewer: Out of 10? Yes.

P12: Oh wait, did you have another question?

Interviewer: That's fine. What about how distressing?

P12: The distress is probably the same.

Interviewer: One to two?

P12: Yes.

Interviewer: How did you feel about watching that one in general?

P12: Fine. That didn't really freak me out or anything. It looks like some nice exercise.

Interviewer: Number two.

P12: Next one?

Interviewer: Yes.

P12: On the second one, the guy on the left looked a little bit like-- He was stopping suddenly and then changing directions really fast which made me think-- I can't remember if the woman was doing the same thing but I noticed he especially. I guess that made me feel like he should be careful doing that because that's how you can get ACL injuries, changing directions really fast. That's what I thought of that one.

Interviewer: How about fear? How would you rate your fear 0 to 10?

P12: I guess I'd probably give it a little bit higher than the one before. Maybe a four or something. The distress, let's say the distress five, that change in direction. Next one?

Interviewer: Next one.

P12: Are these all the same? Just slowed down, is that the key?

Interviewer: No, they're all different. Number three, yes.

P12: They've both got the injury. [laughs] I shouldn't be laughing. I don't know why I'm laughing. I feel upset for them if they've hurt their knee seeing that. The fear, I'd probably only give the fear maybe a six because it could have just been a little sprain which isn't the end of the world. I guess it's more distressful rather than fearful having something happen to your knee. Probably it'd be more distressful, like a seven or something in that scenario. The last one.

Interviewer: How do you feel about that one?

P12: That last one was bad because that looks like how I did my knee kind of, how he jumped and landed on it, not as bent. I will put these ones as a 9 to 10 fear and also same for distress. I hate seeing ACL injuries. Those were a lot worse watching than these other ones.

Interviewer: Does that make you think about your injury when you see that?

P12: Yes. Actually I was waiting for one that looks exactly like mine. Of course, it popped up at the end.

Interviewer: Did you have any other feelings or thoughts about that last video in particular?

P12: It gives you bad memories and makes you not want to play sport again, I reckon. Watching a compilation of ACL injuries especially the way that you did your one makes you think maybe you shouldn't go back to playing netball again because that's what's going to happen or that's what could happen.

Interviewer: Okay. Thanks. Excellent. Not excellent but excellent answers.

P12: [laughs]

Interviewer: Thanks so much for today. Have you got any other thoughts or feelings around the questions or our chat or anything?

P12: Not really. If there's anything that I said today that you go back and look on and you want to probe a little bit more, I'm happy to try and give you a bit more in writing because then at least it gives me a bit of time to reflect a bit more, or try and articulate something a bit better. If there's anything you need a little bit more of, please let me know. I'm happy to help.

Interviewer: Awesome.

[END OF AUDIO]

Participant 13

Interviewer: Okay. Thanks for that. Alright, so now I am going to show you a series of videos of people performing activities that are going to increasingly load their knees. So if you are feeling overly distressed by this. Let me know. I'll stop the video immediately. After each video I'm going to ask how you feel about this video and to rate your levels of fear and distress. Does that sound okay?

Interviewer: Okay. So how did you feel about that video?

P13: Not too bad like a little bit little bit anxious but not really, actually not it was fine.

Interviewer: Okay, so out of a rating 0 to 10 with 0 no fear at all and 10 exteremely fearful. How would you rate

P13: Maybe a 2.

Interviewer: What about zero no distress at all and 10extremely distressing. How would you rate it?

P13: Probably a 0

Interviewer: So into the next video, so number two

P13: so if I'm thinking if I had to do this? these actions?

Interviewer: how do you feel about watching my video watching that action?

P13: I wouldn't want to do it but it's like it's okay watching someone else do it.

Interviewer: Okay, so out of zero no fear at all to 10 extremely fearful how would you rate that one.

P13: Maybe like a three

Interviewer: Yeah, and what about how distressing, 0 no distress at all to 10 extremely distressing

P13: like a one.

Interviewer: Okay. Video three.

P13: Okay, it make me nervous watching all these, actually looks like it's gonna get worse.

P13: Okay. Yeah they fell over, Yeah, it was distressing.

Interviewer: So how do you feel about watching that video.

P13: Yeah, like it brought on a little bit of anxiety.

Interviewer: Zero being no fear at all, to 10 being extremely fearful.

P13: um When you say fearful is that in relation to what I would do because if it's in relation to what I would do probably be like a seven or an eight. You maybe like a seven or eight

Interviewer: Okay.

P13: but like yeah if I'm thinking about me doing it, I would not do that. Yeah, but distressing wise.

Interviewer: yeh, zero no distress at all to 10 extremely distressing.

P13: Yeah, probably about an eight.

Interviewer: What makes it so distressing and fearful?

P13: Just the fact that it looks like they've hurt their knee. It just yeah a little bit traumatic because they fall over and grab their knee.

Interviewer: Okay last video you can watch as much or as little of this one as you want.

P13: No, this one really - oh my God. Okay, that's enough. That's enough okay no more.

Interviewer: So how did you feel about that last video?

P13: I did not like that.

Interviewer: Okay, how did you feel, zero no fear at all? To 10 extremely fearful.

P13: now when I watch that one, I feel like yeah, that's pretty high. Probably a 10.

Interviewer: Okay, and how distressing, zero no distress at all to 10 extremely distressing.

P13: Probably about a 10,

Interviewer: So did any of these videos bring on similar feelings or emotions to kind of what you experienced during your ACL injury Journey.

P13: Yeah.

Interviewer: in what way?

P13: um, I don't know. I guess just the mechanism of it like I don't know.

Interviewer: So being confronted by it

P13: Yeah confronted. Yeah, definitely. That's a good word. Yeah, it's quite confronting because that's probably what I looked like not in all situations, but you know in some way Yeah.

Interviewer: So you identify, you look at the action and you immediately kind of think oh, that's you know, that's what I went through.

P13: Yeah. Yeah, pretty much. Yeah.

Interviewer: Okay.

P13: Yeah, like I cant really explain it but yeah, I definitely identify and exactly what you said. It's quite confronting. Yeah, yeah.

Interviewer: How would you describe the feeling when you watch those videos, especially maybe the last two?

P13: I'm not sure. I guess I know I feel quite uneasy just doesn't I don't know. So Im not to sure

Interviewer: Uneasy is a good descriptor word. Thank you so much for today, have you got any other comments or any thoughts about interview or anything else you'd like to add.

P13: That was fine. I'm sorry went through a bit longer than anticipated just probably because I had lots of injuries.

Interviewer: I'm really appreciate your time. I'll just stop the recording.

Participant 14

Interviewer: Okay. I'll just show a series of videos of people performing activities that load their knees. If you feeling overly distressed by this, let me know. I'll stop the video immediately. For each video, I'm just going to ask you how you feel about the video, and then to rate your levels of fears 0 to 10; 0 being no fear at all, to 10 being extremely fearful. Then, also distress 0, no distress at all, to 10 being extremely distressing.

P14: Okay. Just to clarify, in terms of fearful, is it fearful for the person in a video? Or is it fearful for myself? Undergoing that action?

Interviewer: Yes. How you feel just looking at the video.

P14: Cool.

Interviewer: All right. I just sent through in the chat, there's four videos with YouTube links. If you just click on the top one, and just play it through, and then when you're done.

P14: All right.

P14: The first one was pretty good. What was it, 0 out of 10 I was fearful, and 0 to 10 out of distress?

Interviewer: Yes. How fearful was it? 0 to 10. 10 the most extreme for you.

P14: A two, not even, a one. One is or two, low. Distress, again, it would be, it'd compliment that, I'd say one or two. Low.

Interviewer: How did you feel about watching that video?

P14: It was pretty good. Knowing that this isn't a conversation about ACLs, I immediately go to, and I'll just track the alignment of the knee. If you were to show me a video of two people, male and female jogging, and turning at the very end, I wouldn't even look for it. I would just like, "Yes, that's great. You're running, you're jogging. Cool, next."

Just knowing that this is about ACL, I am looking at the knees and I'll just look at their technique comparing it to mine. I don't feel anything other than looking at their technique. I don't feel any level of stress. I didn't feel if I was to do that if I was in their position that I would be alarmed by anything. No negative thoughts, I'm only just analyzing their technique, if anything.

Interviewer: Have a go number two.

Interviewer: How did you feel about that video?

P14: How did I feel? It was pretty good. The cutting put me a bit uneasy compared to the other one, but it's still fine. I would do that okay.

Interviewer: 0 to 10?

P14: sorry, go ahead.

P14: 0 to 10 being fearful? [silence] Three. Three fearful and the distressed one I would say, three again. Going back to how I'm feeling, I'm just comparing myself. I don't feel too bad, it's all good.

Interviewer: Video number three?

P14: Oh, that's no good. [silence]

Interviewer: How do you feel about that one?

P14: Seeing them hurt themselves puts me uneased. The more I see people getting hurt, doing actions which could possibly hurt my knee, or reinjure my ACL, that puts me a bit uneasy. I would put that like a five or a six. That would be up there a lot. More medium, five or a six. I feel like I could definitely do that, especially in the sport that I do, with the frisbee and the nature of running and cutting almost every ten seconds like that. Seeing them go down, I could almost picture myself in that position. So I'd say six.

Interviewer: Six fearful. Is it six distressing as well?

P14: Distressing wouldn't quite- a five, I'd say. I'm more fearful of doing it myself than I feel distressed if that makes sense.

Interviewer: Video number four?

[silence]

P14: Oh my god.

P14: I'm going to put that one right up there. That's a 10 fearful. That's what I think at night, every-- If I was to play sport the next day. If I was to return to sport-- Everything I just saw there is things that are just replaying in my head, things that I do not want to happen. I'm very fearful that, just watching that, just ugh. That's bad, that's really bad. I think that's just the challenges of doing an ACL, to me, just to always second-guess myself whenever I take the playing field. To see stuff like that but, even worse, happening to me. That's 10 for sure.

Interviewer: What about how distressing? 0, no distress, to 10, extremely distressing.

P14: [silence] I'd say eight. The main things I find distressing there is- I look at it in two ways, for the athletes in the picture, but also for myself. For the athletes, you just see that and you say, "Well there goes the next year of your career." It's the trauma, the injury, but also how it affects you, how it affects your livelihood, and whatnot. That's for the athlete. For myself, very similar thoughs but more so about, because I've done it in the past, and I've done it again, just knowing that if I take the sporting field again, I'm even more susceptible to re-doing it.

It always comes back to return to sports for me. Already being on edge as I am, doing that and then just knowing that, "All right, well, should I try and give up competitive sports?" That's the way I think. I just don't want to do it. From the pain associated with it, the rehab and the time it takes and, like I've touched on, the financial implications of it as well. I always sympathize with the athletes when I see someone go down, whether that's a professional, or whether that's an amateur. I always just end up comparing myself to everyone, whether that's comparing in the rehab stage or comparing

when someone does it. I just always think, "Shit, that could be me." I could've landed awkwardly like that, or I could've stepped like that, and my knee would've buckled.

Interviewer: You mentioned that as well. You said, "things that you think about at night." Do you think about knee-related problems at night?

P14: No. Only if I was doing sport the next day. That's, again, because lockdown has chained me from jumping back into sports it's only happened once, but it hasn't happened since then. It's just a flicker of seeing an athlete jump for a rebound and then on the landing, his knees buckled. I just get little flashbacks of that. It's bad, but it's not too bad, if that makes any sense. It's just reliving my injury. Like I said, it's only happened once. I could hit you up again when it happens again. Like I said, because I haven't had that opportunity to play sports often since like 12 months, since getting in the old "all clear" to return back to sport.

I can't really talk on it too much. I'm happy to have this as a reoccurring conversation, if you will. If I do get back into competitive sport, happy to share my thoughts and feedback about the day before sport, the day of sport, and then after the activity. To answer your question, it's only happened once, that triggers that flicker in my mind.

Interviewer: Triggers the flicker in your mind? As in- explain that a little bit more to me?

P14: Yes, definitely. Like if I know that I'm doing something that I classify as at risk. The best example for me is if I knew I was kicking a footy

01:09:49

like the first time I went back to kicking a footy I would often-- Sorry, not often. It's only happened very rarely but I would just have like little flashes of seeing like a Dale Morris go down with his knee, or a Bob Murphy. I'm a Bulldogs fan by the way. Just seeing people that I've seen that I enjoy watching, or seeing anyone, for that matter, just redoing their knees. The only ones that come to mind is that Bob Murphy, Dale Morris because they've happened quite recently.

I shouldn't say it really, but the first time I do a particular activity-- for example, the first time I kicked a footy, I did see little flashes of Bob going down doing his knee because he landed that way. Then I would steer away from that so when the ball would come and I would jump up as I normally would, I think I landed on the other good leg to support it before cushioning it with my left, my injured leg. That was for footie. That was the first time I went back on to and kicked to the park.

The first time I was throwing the frisbee but it was more like intense kind of a-- that involved actually running and cutting back. Before I did that, again I had these little flashes seeing someone run, change direction, and go down. Then I just know that this is what I'm playing with so when I do cut, I would initially cut on my not injured leg, and then the second time I'd do it, I'd try to do it on my injured leg. It's almost like as if I'm-- it comes unintentionally which is not good. I always look at it in a bright way by saying, "Okay, that's how this person went and done it. Let's not do that." It only happens the first time I ever do a particular movement.

Then after that, I go out on the-- you might tell me I go out and kick a footie, I wouldn't have those flashbacks. It only ever happens the first time I go back out there. I think it's just because I get scared, I get nervous, my body just goes, "Hey, you're doing this again. If you do this wrong, this will happen" and then it shows me that. That's the footie example, that's a frisbee example.

A basketball example is when we were just shooting on the first time I went out onto the court. It was just really, really casual but I did, I would see someone take a rebound, land, screw up their knee, and then-- I don't know if that's to make my body try to trick me to not do it. I fight through it. It only happens the first time.

They were the only three that had really stood out to me. Everything else was-- Look, if I had known that I was going to have this conversation, I probably would remember it more because it's all in hindsight and you're asking me to recall it. It hasn't been too bad in terms of, now it's picking up. I guess in hindsight that's probably a bad thing. You're probably having red flags in your mind, just hearing me say all this and that.

In all honesty, it was very lighthearted, it was just a way of-- almost it's psyching myself up in terms of, "You'd better use right technique here…

or else you're going to screw up again." At least that's how I like to look at it. I don't have to look at it as a way that I'm traumatic but I'm getting all these traumatic flashbacks. I almost treat it as if my body's telling me that, "Hey, you don't have the best knees at the moment. Do it right or else this will happen." That's how I see it.

Interviewer: Do watching those videos bring on some of those emotions?

P14: That last one did, definitely. Yes, that one did because-- That last one did, definitely. That wasn't good. I don't think I could watch it over and over again. Every time I see someone go down with a knee, it doesn't even have to be an ACL but I feel my knee twitch. If I see someone go down holding their knee, I would sometimes hold my knee, as well.

I don't know if that happens in you as well, but if I see someone go down with their knee, I just give my knee a bit of a rub. I don't know how else to put it. I don't ever feel pain seeing someone else do their knee. I don't feel pain in the knee, that is. Obviously, I feel pain, I feel stressful, I get anxious when I see someone go down screaming in agonizing pain holding their knee knowing that it may be an ACL. I feel that pain but that's only in terms of that stress, anxiety whatever. I never feel a painful knee, but I do grab at it if I see someone lunging and grabbing at their knee. It's weird.

Interviewer: Awesome.

Participant 15

Interviewer: Okay. All right. Well, that's great. Great chat. I should say, not great, your experience. I'm just going to show you a series of videos of people performing some activities that increasingly load the knees. If you're feeling overly distressed by this, let me know. I'll stop the video immediately. After each video, I'm going to ask you how you feel about the video. I'll say, "How do you feel?" Then I'll say, "How fearful are you?" Zero to 10. Zero being no fear at all. 10 being extremely fearful. Then I'll also ask the same for distress. How distressed are you? Zero being no distressed, 10 being extremely distressed. Watch the clip the whole way through and then tell me how you feel first. Does that sound okay?

P15: Yes, sure thing. I watch all four, and then?

Interviewer: Just watch the first one, and then we'll chat about that. Then, the second one.

P15: All right.

[pause]

P15: Cool. I've watched the first one.

Interviewer: How do you feel about that one?

P15: Fine. It's people running and then turning around. I guess it was pretty slow. Pretty controlled. You can see all the muscles working. Zero, I guess.

Interviewer: Zero for fear. What about distress?

P15: For fear and zero for distress.

Interviewer: All right. Next video.

[pause]

P15: Cool. All done.

Interviewer: How did you feel about that one?

P15: It was all right. I was wondering what they were going to do. Was it just a plant and cut. It was pretty straightforward. Although, it was fine. I'm just not sure. I'm waiting for their knees to just actually blow out, [chuckles] considering the context of the discussion obvious, but yes, pretty fine.

Interviewer: Is there any fear? Fear, 0 to 10, where would you rate it?

P15: Fear, probably one because he bended his knee a little bit, and I got a little considering the context of the discussion. Distress, probably one.

Interviewer: Next video.

[pause]

Interviewer: How do you feel about that one?

P15: Not great. I don't want to see anyone clutch their knee and fall down, especially after hyperextending. Yes, not great. You know it just looks innocuous that you don't know what's going on underneath there. Probably about a three, I guess, because it's a pretty straightforward turn, the

degree of difficulty is not that high. Distress is probably at a four or a five or so because you don't want to see anyone get injured at all, especially in the knees.

Interviewer: Thanks. Last video.

[pause]

Interviewer: How did you feel about that one?

P15: It's a bit more real. It's a bit more real. You don’t like seeing people tear their ACLs, I didn't know people could tear their ACLs doing javelin anyway. [laughs] They're all elite athletes but it's like, "Shit, that's their livelihood." For me, in terms of fear, it doesn't make me feel super fearful because you see sports stars blow their knees out all the time, and they've got the best of facilities and recovery and things like that. I've seen them either limping or falling, it wasn't them being absolutely crunched, if that makes sense. Not to say that I was. In terms of distress theres a few of them that's make you go, "Oh." Theres a few that make your breath go, "Oh, shit." It sounds like fear is probably a three, end distress is probably a six or a seven or so. You don't want to see anyone's knee bend that weird. It's doesn’t show them going down, or up where it felt like.. but it doesn't really show you them holding their knee, and afterwards that second of them thinking that, "Oh, shit, my life is going to change considerably after this one second for the next few years. My life choices have changed and my options are limited."

Interviewer: A lot's occurring in that second of after you go down.

P15: That whole minute/10 minutes/what are you going to do? You're trying to get all your affairs in order, and you think, "Shit, I have to get organized. What do I have to organize to be prepared?" At least what's what I think about it.

Interviewer: Did any of these videos bring about similar emotions or feelings than when you experienced your ACL injury?

P15: Yes. The immediate reaction that I have sometimes when you see other people tear their ACL is you want to go, "Oh, shit." You physically check, "Is my knee still intact?" [laughs] It's something that I go, "Oh, I've felt that pain before, I understand what they're going through." It's a phantom pain that you remember because it's not that I've experienced that many super painful things in my life.

I consider that to be probably number one in terms of pain, and the type of pain and the exact feeling of feeling my knee go over-- Feeling my joint absolutely just give way. It's pretty messy, it's not fun. You're like, "That person's really good," and then being injured, and it's like, "Oh, shit. That person's life is going to change forever now." It's a pretty big deal.

Interviewer: Any other thoughts or comments about the chat today?

P15: No, it's been really good and super reflective for myself at least. That's very helpful.

Interviewer: Good, I'm glad.

[END OF AUDIO]

Participant 16

Interviewer: All right. Thanks for that. Now, I'm just going to show you a series of videos of people performing activities that increasingly load the knee. If you feel uncomfortable about this, let me know and I'll just stop the videos. After each video, I'll ask you how you feel and then how fearful you are on a scale of 0, no fear, to 10, extremely fearful; and how distressed you are, 0, no distress to 10, extremely distressed. Are you ready to proceed?

P16: Ready.

Interviewer: Ready and willing. Awesome. I'm just going to copy and paste into the chat four videos. They're in sequence. We can play them, one, watch it all the way through. When you're ready, we can do that.

P16: So I just click on number one

Interviewer: Yes. Just click on number one.

[pause]

P16: Let me see. For the first one, I would say zero on both for most of the video, but maybe 1 when they turned.

Interviewer: How do you feel about that in general?

P16: Fine. That's not a stressful thing to watch.

Interviewer: Okay. Let's play number two.

P16: Yes.

[pause]

P16: Again, fine and then when they turn-- Similar to the first one, just a very mild, "Ooh."

Interviewer: How fearful from 0 to 10?

P16: Probably a one again. That wasn't a stressful thing to watch.

Interviewer: How distressing, 0 to 10?

P16: Zero to one.

Interviewer: Okay. Video number three.

[pause]

Interviewer: How do you feel about it?

P16: maybe a two.

Interviewer: How fearful, 0 to 10?

P16: I don't know if it makes me feel fearful. I'm not sure whether it's out of fear or distress. It's like an empathy reaction, "Aargh," but it's not personally-- It's not creating a lot of fear for me personally or even distress but, like I said-- That is harder to watch than the first two but not very much. Not so much of a personal impact as the-- Sorry, this is not helpful. [laughs] Giving you a number. I guess in recognition that it was slightly harder to watch than the other three, maybe a two.

Interviewer Two for fear. What about the distress, 0 to 10?

P16: I guess maybe the same a 2.

Interviewer: Last video.

[pause]

P16: It's much harder to watch.

Interviewer: How do feel about that one?

P16: Sorry?

Interviewer: How do you feel about that one?

P16: It feels mildly uncomfortable to watch. It's not something that I would say is distressing, but maybe around a four. Maybe like a four in fear and a three on distress. Not upset by it. It is just that visceral reaction where you just get a little--

Interviewer: Did any of those videos-- You said I think already, but they didn't have a direct relationship with what you were feeling during your injury?

P16: No.

Interviewer: No? Thanks for that today. Amazing. Heaps of good information. That's helped a lot.

P16: That's fine. Good luck with your research.

[END OF AUDIO]

Participant 17

Interviewer: I'll just quickly put them there. There's just a series of people performing activities increasingly of the knees. If you're feeling overly distressed by this, let me know and I'll stop the video immediately. After each video, I'm just going to ask you how you feel about the video and then to rate your levels of fear 0 to 10. 0, no fear. 10, extreme fear; and then, distress. 0, no distress, to 10, extreme distress. Does that sound okay?

P17: Okay, cool. Do I press the link and watch it on [crosstalk]-

Interviewer: You just watch the first one-

P17: -on my thing?

Interviewer: Yes. Just click the first one, watch it through, and then let me know how you feel about it and fear and distress.

P17: Oh, God, when they turn [laugh]. Yes, the first one's through.

Interviewer: How do you feel about that?

P17: I feel like I'd look at the girl more [chuckles] because I see myself. I wouldn't call it distress but I see myself being in her role and I wouldn't move. I wouldn't turn that, really [laugh].

Interviewer: Zero [crosstalk]--

P17: [crosstalk] The level of distress, I would say maybe a three?

Interviewer: Three, distress? Yes? What about 0 to 10, how fearful?

P17: Of me doing that exercise?

Interviewer: Just watching the video and how you feel about it.

P17: Oh. I feel fearful, probably just two just watching it. The thought of doing that is more fearful.

Interviewer: Okay. Two. And then what about the next video?

P17: Let me click the link.

P17: I feel like I'm cautious watching them. I'm fearful of thinking of myself doing that? I'm not necessarily super fearful watching it. someone actually doing that [laugh] on the video, I would be fearful. I think similar the first one, the same thing. I think of myself doing it and--

Interviewer: So is it [crosstalk]-

P17: It's probably the same--

Interviewer: -0 to 10?

P17: -Three, yes. There you go.

Interviewer: 0 to 10, how fearful would you be?

P17: [silence] I think a two or a three?

Interviewer: What about 0 to 10, how distressed would you be?

P17: Three?

Interviewer: Okay. Video number three?

P17: Yes.

P17: No. Whoops, that's mean [laugh].

Interviewer: How do you feel about that one?

P17: Fearful, less. Probably four but distress-

Interviewer: How distressed--

P17: -probably five.

Interviewer: Five? So 4 out of 10 for fear and 5 out of 10 for distress?

P17: Yes.

Interviewer: How do you feel about that one?

P17: As I told you before, I really don't like if I see it on football or something like that, maybe? Because I know it's maybe more of a you're showing it to me kind of thing? So it's a little bit less shocking. But if for example, if before I was watching that, it was football and now it's actually the thing. Sometimes I can't even watch it.

Interviewer: I'll get you to watch the last one but you can just pause it or stop it when you want to, if you need to [silence].

P17: Oh, God. Oh, that's just terrible.

P17: Oh. That was terrible.

Interviewer: How are you feeling about that one?

P17: Fearful in a sense for them, like a seven. But I don't like the stressing out. That's more like a seven. I don't like watching that.

Interviewer: How does it make you feel watching that?

P17: I feel like I just remember the exact feeling of it. The classic empathy kind of thing. I remember the exact feeling. How terrible it was. When I see those videos, I actually think more of the noise? I distinctly remember the noise? When I see those videos, I almost see the noise.

Interviewer: Okay. Thanks so much for that today. Any other comments or thoughts about the interview we just did?

P17: No, but I hope you can do something good with it [laugh]?

Interviewer: All right. Thanks.

[END OF AUDIO]
